# Quality of life after conservative versus elective surgical management of first-episode uncomplicated diverticulitis: a single-center prospective study

**DOI:** 10.1007/s10151-026-03407-4

**Published:** 2026-08-18

**Authors:** G. Quero, L. Langellotti, A. P. Tortorelli, V. Tondolo, B. Biffoni, G. Daloiso, D. De Sio, M. Covino, F. Taglioni, O. Scrima, M. C. Blasi, M. Aulicino, C. Fiorillo, S. Alfieri, R. Menghi

**Affiliations:** 1 Digestive Surgery Unit, Department of Surgery, Fondazione Policlinico Universitario “Agostino Gemelli”, IRCCS, Largo Agostino Gemelli, 8, 00168 Rome, Italy; 2 Dipartimento di Medicina e Chirurgia Traslazionale, Università Cattolica del Sacro Cuore di Roma, Rome, Italy; 3 General Surgery Unit, Fatebenefratelli Isola Tiberina—Gemelli Isola, Rome, Italy; 4 Department of Emergency Medicine, Fondazione Policlinico Universitario Agostino Gemelli-IRCCS, 00168 Rome, Italy

**Keywords:** Diverticular disease, Quality of life, DV-QoL, Uncomplicated diverticular disease, Elective sigmoidectomy, Diverticulosis

## Abstract

**Background:**

Optimal long-term management after a first episode of uncomplicated acute diverticulitis (AD) remains debated. Quality of life (QoL) is increasingly recognized as a key outcome to guide individualized decision-making between clinical surveillance and elective sigmoid resection.

**Methods:**

This single-center prospective longitudinal observational study enrolled adults with a first episode of uncomplicated AD (Hinchey 0-1a). After recovery from the index episode, patients underwent either clinical follow-up (nonsurgery group) or elective laparoscopic sigmoidectomy (surgery group) according to routine clinical decision-making. Diverticulitis-specific QoL was assessed using the DV-QoL questionnaire, retrospectively referring to the month before the index episode and prospectively at 1, 6, and 12 months after post-episode management. The primary aim was to describe and compare DV-QoL trajectories between groups. A DV-QoL score ≥ 3.2 was used to define a non-acceptable symptom state (Patient Acceptable Symptom State (PASS) threshold). Predictors of non-acceptable QoL were explored in conservatively managed patients.

**Results:**

A total of 137 patients were included: 96 (70%) in the nonsurgery group and 41 (30%) in the surgery group. Baseline characteristics were comparable. Pre-episode DV-QoL scores were similar between groups (0.19 ± 0.29 versus 0.12 ± 0.33; *p* = 0.14). DV-QoL total scores were significantly higher (worse QoL) in the nonsurgery group at 1 month (2.60 ± 2.70 versus 0.11 ± 0.15; *p* < 0.001), 6 months (2.09 ± 2.24 versus 0.05 ± 0.12; *p* < 0.001), and 12 months (1.92 ± 2.07 versus 0.02 ± 0.07; *p* < 0.001). Non-acceptable QoL (PASS ≥ 3.2) occurred only in the non-surgery cohort at all timepoints, whereas no surgical patient exceeded PASS. Younger age independently predicted non-acceptable QoL in conservatively managed patients.

**Conclusions:**

Elective laparoscopic sigmoidectomy after a first uncomplicated AD episode was associated with sustained improvements in disease-specific QoL compared with clinical follow-up. Routine DV-QoL assessment may help identify patients at risk of persistent impairment and optimize individualized surgical decision-making.

**Supplementary Information:**

The online version contains supplementary material available at https://doi.org/10.1007/s10151-026-03407-4.

## Introduction

Diverticulosis is an asymptomatic clinical condition affecting approximately 50% of individuals over the age of 60 years in the USA [1, 2]. According to recent studies, about 5% of patients with diverticulosis will develop acute diverticulitis (AD) over time, defined as an inflammatory process of the colon resulting from inflammation of diverticula with or without associated complications [1–3]. This condition accounts for nearly 300,000 hospital admissions annually, with an estimated economic burden of approximately 9 billion dollars on the national healthcare system [4, 5]. From a clinical standpoint, AD can be categorized into uncomplicated and complicated forms [1, 6]. The uncomplicated form is characterized by acute inflammation of the colonic wall and pericolic fat, in the absence of radiological or clinical signs of abscess, perforation, fistula, or stricture. In contrast, the complicated form refers to diverticular inflammation associated with one or more complications, including pericolic abscess, perforation, fistula formation, stricture, or diverticular bleeding [6–10].

The uncomplicated form of AD accounts for approximately 75–85% of all cases of diverticulitis [6].

In accordance with contemporary international guidelines [11–15], uncomplicated AD is primarily managed conservatively. Treatment should be tailored according to clinical severity, immune status, and comorbidities. In immunocompetent and clinically stable patients, outpatient management and selective rather than routine antibiotic therapy are currently recommended. However, although most patients experiencing a first episode of uncomplicated AD do not develop further episodes, approximately 8% have a risk of recurrence within 1 year, 19% experience a second recurrence, and 24% will have an additional episode after the third recurrence [3, 6–10]. This evidence continues to fuel the debate in the literature regarding the optimal management strategy after an episode of uncomplicated AD, by evaluating the potential benefits of elective surgical treatment following the resolution of the first episode of acute uncomplicated AD. Previous recommendations supported elective surgical resection only after multiple episodes of AD or in cases of acute inflammatory diverticular disease occurring at a young age [13]. However, contemporary guidelines [11–15] have moved away from episode-count-based indications and recommend individualized decision-making when elective surgery is considered. Importantly, current evidence does not support routine elective resection after a first episode of acute uncomplicated diverticulitis. Instead, surgery may be discussed in selected patients after careful assessment of symptom burden, quality of life (QoL), and operative risk.

In this context, the assessment of QoL is progressively gaining a predominant role, representing a key element in defining the overall impact of the disease on the patient’s daily life and serving as an essential tool to determine the most appropriate management strategy [16].

On the basis of these premises, the aim of this prospective study is to evaluate the QoL of patients following a first episode of uncomplicated AD treated conservatively, and subsequently managed either with clinical follow-up or elective surgical resection, to determine the most appropriate therapeutic strategy.

## Materials and methods

### Study design and data collection

This was a single-center, prospective, longitudinal, observational study conducted at the Fondazione Policlinico Universitario Agostino Gemelli IRCCS of Rome between January 2021 and December 2023. The study was approved by the Institutional Ethics Committee (ID: 4522 Prot N. 0030679/21) and carried out in accordance with the Declaration of Helsinki. Written informed consent was obtained from all individual participants included in the study.

Only patients aged 18 years or older who were admitted to the Emergency Department or the Digestive Surgery Unit of the Fondazione Policlinico Gemelli IRCCS with a clinical and radiological diagnosis of a first episode of uncomplicated AD were prospectively enrolled during the index episode and subsequently followed according to the study protocol. Exclusion criteria were previous history of emergency surgery, previous abdominal operations, prior episodes of acute diverticulitis, and/or diagnosis of inflammatory bowel disease.

AD was graded according to the modified Hinchey classification [13]. Accordingly, only patients with grades 0 and 1a (corresponding to uncomplicated AD) were enrolled in the study. All recruited patients underwent conservative treatment according to the current guidelines [11–15] until complete resolution of the clinical condition. After resolution of the index episode, patients entered routine post-diverticulitis management and were classified according to the strategy actually undertaken: clinical follow-up or elective sigmoidectomy. Treatment allocation was not protocolized and was not randomized. In detail, after complete resolution of the index episode, patients were reassessed during outpatient follow-up, which at our institution is routinely scheduled approximately 1 month after discharge. During this visit, clinical recovery, symptom resolution, and subsequent management were discussed within routine clinical practice. Elective sigmoidectomy was not routinely proposed after the first episode of uncomplicated AD. The decision to proceed with surgery was based on individualized shared decision-making between the treating surgeon and the patient. Although no protocol-defined criteria were adopted, factors considered included persistence of diverticulitis-related abdominal symptoms, impact on daily activities, dietary or behavioral limitations, anxiety regarding recurrence, occupational or social impairment, and patient preference after detailed counseling. Patients were explicitly informed that conservative follow-up represents the standard management after a first uncomplicated episode, and the potential benefits of surgery were balanced against operative and long-term risks. All patients considered for surgery underwent standard preoperative counseling and provided written informed consent for the surgical procedure.

The following clinical–demographic characteristics were prospectively collected: age, sex, body mass index (BMI), smoking status, and comorbidities (also stratified for Charlson Comorbidity Index (CCI) [17]). For the surgery group, additional perioperative variables were retrieved, including surgical approach (open or laparoscopic), operative time, need for ostomy, postoperative complications (including anastomotic leakage), postoperative mortality, and length of hospital stay. Postoperative complications were graded according to the Clavien–Dindo classification [18], while postoperative mortality was defined as any death occurring during the hospitalization. Anastomotic leakage was defined and classified according to the International Study Group of Rectal Cancer (ISGRC) classification [19].

### Study outcomes

The primary outcome of the study was to assess and compare QoL between patients who underwent clinical follow-up (nonsurgery group) and those who underwent elective laparoscopic sigmoidectomy (surgery group) after a first episode of uncomplicated AD (Hinchey 0–1a) managed conservatively.

### Quality of life assessment

#### The Diverticulitis Quality of Life questionnaire

The Diverticulitis Quality of Life (DV-QOL) instrument is a disease-specific tool designed to assess the impact of AD on patients’ QoL. It includes 17 items distributed across 4 domains: physical symptoms (5 items), concerns (3 items), feelings (4 items), and behavioral changes (5 items) [20–22]. For the nonsurgery group, DV-QoL was collected at 1, 6, and 12 months after the index diverticulitis episode. For the surgery group, DV-QoL was collected at 1, 6, and 12 months after elective sigmoidectomy. Therefore, follow-up assessments were anchored to different post-episode milestones across groups rather than to a common time origin, and comparisons should be interpreted as reflecting QoL after the management strategy actually undertaken rather than strictly time-matched outcomes after the index episode. This reflects real-world decision-based outcomes rather than strictly time-aligned comparisons. To assess the baseline gastrointestinal status before the first episode of AD, all patients were also asked to complete the DV-QoL questionnaire referring to 1 month before their first episode. This baseline assessment was administered at the first scheduled follow-up visit, approximately 1 month after recovery from the acute episode.

#### Scoring

Each domain score is derived by converting the sum of item responses into a 0–10 normalized scale, where lower values reflect better quality of life. The overall DV-QOL score is calculated summarizing the four domain scores, with each domain contributing equally, as described by Khor et al. [23]. A Patient Acceptable Symptom State (PASS) threshold of 3.2 was established: scores < 3.2 correspond to an acceptable symptom burden, whereas scores ≥ 3.2 indicate symptom levels associated with a clinically relevant reduction in QoL [23].

### Surgical and nonsurgical treatments

All patients included in the study underwent conservative management individualized according to clinical condition, comorbidities, oral intake tolerance, and the presence of systemic signs of inflammation or infection. Supportive care, dietary modification, intravenous fluids, and antibiotic therapy were administered selectively when clinically indicated, in accordance with contemporary guideline recommendations [11–15]. Per protocol, at discharge, all patients were prescribed a standard therapy consisting of oral neomycin and probiotics, to be administered in 1-week cycles per month, regardless of any recurrence of symptoms, together with a disease-specific diet for diverticular disease.

Patients in the nonsurgical group underwent clinical follow-up, while patients in the surgical group were electively scheduled for laparoscopic sigmoidectomy. The procedure followed the standard steps of sigmoidectomy, including isolation and division of the sigmoid vessels, distal resection at the rectosigmoid junction using a triple-row linear stapler, proximal transection at the descending colon–sigmoid junction, and end-to-end colorectal anastomosis using a triple-row circular stapler. In all cases, a hydro-pneumatic leak test of the anastomosis was performed, and the creation of a protective ileostomy was left to the discretion of the operating surgeon or in case of positive hydro-pneumatic leak test.

Postoperatively, adequate analgesic therapy was administered throughout the hospital stay, along with supportive intravenous fluid therapy for the first 12 h. Patients resumed a liquid diet 24 h after surgery, progressing to regular oral intake according to the clinical conditions. Laboratory tests, including blood chemistry and complete blood count, were performed on postoperative days 1, 3, and 5. Discharge occurred upon restoration of regular oral intake and bowel function (passage of gas and/or stool), in the absence of fever or abnormalities in laboratory findings.

### Statistical analysis

Continuous data were reported as mean ± standard deviation (± SD), and categorical data as number and percentages. Univariate analysis was performed with Student’s *t* tests, Mann–Whitney *U* tests, Fisher’s tests, and *χ*^2^ test. For all analyses, a *p*-value ≤ 0.05 was considered as statistically significant. Post hoc statistical power was calculated using the observed effect sizes derived from paired mean differences (Cohen’s d) for the primary endpoint. Power estimates were obtained using standard analytical methods under *α* = 0.05 and number of patients involved.

To explore potential nonlinear associations between age and QoL in relation to study outcomes, QoL was modeled using restricted cubic splines. Expected QoL values were derived from the beta coefficients of multivariate linear regression models and plotted against age. Adjusted predicted QoL at 6 and 12 months was estimated from model-based predictions and displayed according to operative intent (resection versus clinical follow-up). A restricted cubic spline (RCS) curve was fitted to describe this relationship, using age as a continuous predictor and adjusted predicted QoL as the outcome. This nonparametric approach was selected to provide a flexible representation of the association without imposing a predefined parametric form. Knots were placed at the 25th, 50th, and 75th percentiles of the age distribution, following standard recommendations for spline modeling. These graphical analyses were intended as descriptive tools to aid interpretation and were not used for inferential testing or hypothesis evaluation.

To identify independent predictors of poor quality of life (DV-QoL ≥ 3.2), we included in a logistic regression model all variables that were significant in the univariate analysis. Since all patients who underwent surgical resection achieved a satisfactory QoL outcome, we performed a sensitivity analysis restricted to patients managed conservatively. Results of the logistic regression analysis are reported as odds ratios (ORs) with 95% confidence intervals.

All tests were performed using SPSS version 25 for Windows (SPSS Inc., Chicago, IL, USA).

## Results

### Demographical and clinical characteristic of the population

A total of 137 patients were enrolled in the study: 96 (70%) underwent clinical follow-up after the first episode of uncomplicated AD and constituted the nonsurgery group, while 41 (30%) underwent surgery after the conservative treatment and constituted the surgery cohort (Fig. S1). Baseline demographic and clinical characteristics of the study population are reported in Table 1. No statistically significant differences were detected between the two study cohorts in terms of sex, age and BMI. Similarly, the two study cohorts were comparable for major comorbidities, except for pulmonary diseases, more frequently encountered in the surgery cohort (3 patients, 7.3%) as compared with the nonsurgery group (1 patient, 1%) (*p* = 0.05). The distribution of Hinchey grades at presentation was comparable between groups, with the majority of patients classified as Hinchey 1a (73 patients, 76% and 31 patients, 75.6% in the nonsurgery and surgery groups, respectively; *p* = 0.95).

**Table 1:** Demographical and clinical characteristics of the study groups

	Population (*n* = 137)	Nonsurgery (*n* = 96)	Surgery (*n* = 41)	*p*
*Sex, n (%)*				
M	60 (43.8)	37 (38.5)	23 (56.1)	0.06
F	77 (56.2)	59 (61.5)	18 (43.9)	
Age, years, mean ± SD	59 ± 13	59 ± 13	66 ± 15	0.97
BMI, mean ± SD	24.5** ± **4	24.4 ± 4	23.5 + 2	0.63
Cardiovascular comorbidities, *n* (%)	67 (49)	45 (47)	22 (53)	0.47
Diabetes, *n* (%)	6 (4.4)	6 (6.2)	0	0.10
Pulmonary comorbidities, *n* (%)	1 (0.7)	1 (1)	3 (7.3)	**0.05**
Charlson Comorbidity Index, mean ± SD	1.63 ± 1.41	1.73 ± 1.54	1.39 ± 1.04	0.13
Smoker or ex-smoker, *n* (%)	48 (35)	36 (37)	12 (30)	0.33
*Hinchey grade, n (%)*				
0	33 (24.1)	23 (24)	10 (24.4)	0.95
1a	104 (75.9)	73 (76)	31 (75.6)	

Bold values indicate statistically significant results (*p* < 0.05)

### Surgery group and postoperative outcome

Intraoperative features and postoperative outcomes of the surgical group are summarized in Table 2. Surgery was performed after a mean time of 3.1 + 1.1 months after the first episode of AD. No AD recurrence was documented before surgery. No intraoperative conversions were recorded. An ostomy was required in one patient (2.4%) for a positive hydro-pneumatic leak test. Postoperative morbidity was low, with 4 patients (9.7%) experiencing Clavien–Dindo grade I–II complications, while no major adverse events (grade III–IV) were observed in any case. No anastomotic leakage or in-hospital mortality occurred. Incisional hernia was reported in two patients (4.9%). The mean length of hospital stay was 6 (**± **2) days.

**Table 2:** Intraoperative and surgical outcomes of the surgery group

	Surgery group (*n* = 41)
*Intraoperative outcomes*	
Operative time, min, mean ± SD	191 (± 68)
Conversion rate, *n* (%)	0
Ostomy, *n* (%)	1 (2.4)
*Postoperative outcomes*	
* Clavien-Dindo, n (%)*	
I–II	4 (9.7)
III–IV	0
Anastomotic leakage, *n* (%)	0
Incisional hernia, *n* (%)	2 (4.9)
In-hospital mortality, *n* (%)	0
Length of hospital stay, days, mean ± SD	6 ± 2

### Nonsurgery group

Of the 96 patients managed conservatively, 14 patients (14.6%) experienced AD recurrence after a mean time of 9 ± 3.2 months after the first AD episode. Among these, 6 (6.3%) patients presented Hinchey grade 0-1a, 6 (6.3%) grade 1b, and 2 (2.1%) grade 4, that required emergency surgical intervention with colostomy formation. The remaining recurrences were managed conservatively without the need for urgent surgery.

### QoL comparison between surgery and nonsurgery group

DV-QoL scores, at 1 month before the AD episode (baseline measure), were comparable between the nonsurgery and surgery cohorts, with a value of 0.19 ± 0.29 and 0.12 ± 0.33, respectively (*p* = 0.14). Conversely, DV-QoL questionnaire scores were significantly lower in patients undergoing surgery compared with those managed only conservatively at all evaluated follow-up timepoints (Table 3). At 1 month, the nonsurgery group reported a significantly higher mean DV-QoL score compared with the surgical group (2.60 ± 2.7 versus 0.11 ± 0.15, respectively; *p* < 0.001), indicating a worse QoL. Similarly, at 6 months, DV-QoL scores remained significantly higher in the nonsurgery group than in the surgery cohort (2.09 ± 2.24 versus 0.05 ± 0.12, respectively; *p* < 0.001). This difference persisted at 12 months, with the nonsurgery patients reporting worse DV-QoL scores compared with those who underwent elective surgery (1.92 ± 2.07 versus 0.02 ± 0.07, respectively; *p* < 0.001).

**Table 3:** Comparison of DV-QoL questionnaire scores at 1, 6, and 12 months between surgery and nonsurgery groups

Timepoint	Nonsurgery group (*n* = 96)	Surgery group (*n* = 41)	*p*	Effect size (Cohen’s d)
Baseline, mean ± SD	0.19 ± 0.29	0.12 ± 0.33	0.14	1.01
1 month, mean ± SD	2.60 ± 2.7	0.11 ± 0.15	< 0.001	1.10
6 months, mean ± SD	2.09 ± 2.24	0.05 ± 0.12	< 0.001	1.08
12 months, mean ± SD	1.92 ± 2.07	0.02 ± 0.07	< 0.001	1.09

Large effect sizes were observed at all timepoints (Cohen’s d > 1.0), indicating a sustained benefit of surgical treatment on disease-related QoL over time.

Overall, the analysis of DV-QoL subdomains showed a robust and sustained advantage of surgical treatment over conservative management at all the follow-up timepoints, with statistically significant differences consistently observed across every domain and individual questionnaire item (Table S1).

Patients were thus categorized according to the PASS threshold (Table 4). At 1 month, a DV-QoL questionnaire score > 3.2 was documented in 32 (33.3%) patients of the nonsurgery cohort as compared with none in the surgery group (*p* < 0.001). This statistical difference was furthermore confirmed at the 6-month (27 patients (28.1%) and 0 patients presenting a DV-QoL score > 3.2 in the nonsurgery and surgery cohorts, respectively; *p* < 0.001) and 12-month (25 patients (26%) and 0 presenting a DV-QoL score > 3.2 in the nonsurgery and surgery cohorts, respectively; *p* < 0001) analyses.

**Table 4:** QoL comparison between nonsurgery and surgery treatments according to the DV-QoL questionnaire PASS threshold

Timepoint	Nonsurgery (*n* = 96)	Surgery (*n* = 41)	*p*
*1-month follow-up*			
DV-QoL score ≤ 3.2, *n* (%)	64 (66.7)	41 (100)	< 0.001
DV-QoL score > 3.2, *n* (%)	32 (33.3)	0 (0)	
*6-month follow-up*			
DV-QoL score ≤ 3.2, *n* (%)	69 (71.9)	41 (100)	< 0.001
DV-QoL score > 3.2, *n* (%)	27 (28.1)	0 (0)	
*12-month follow-up*			
DV-QoL score ≤ 3.2, *n* (%)	71 (74)	41 (100)	< 0.001
DV-QoL score > 3.2, *n* (%)	25 (26)	0 (0)	

### Multivariate analysis of prognostic factors for PASS threshold ≥ 3.2 in the nonsurgery group

A multivariable analysis was performed to identify factors associated with unacceptable QoL (PASS ≥ 3.2) in conservatively managed patients (Table 5).

**Table 5:** Multivariate analysis of factors associated with non-acceptable DV-QoL (≥ 3.2) in the nonsurgery group

Variables	1-month PASS threshold		*p*	Multivariate analysis odds ratio [95% confidence interval]	*p*
	< 3.2 (*n* = 64)	≥ 3.2 (*n* = 32)			
*Age*					
< 57	27 (42)	24 (58)	**0.002**	0.28 [0.11–0.71]	**0.04**
≥ 57	37 (58)	8 (42)			
*Sex*					
M	26 (40)	11 (35)	0.55	–	–
F	38 (60)	21 (65)			
*BMI*					
< 24	34 (53)	13 (46)	0.23	–	–
≥ 24	26 (46)	17 (53)			
CCI					
< 1	27 (42)	22 (68)	**0.01**	0.83 [0.22–3.12]	0.78
≥ 1	37 (58)	10 (32)			
*Hinchey grade*					
0	14 (22)	9 (28)	0.49		
1a	50 (78)	23 (72)			

Variables	6-month PASS threshold		*p*	Multivariate analysis odds ratio [95% confidence interval]	*p*
	< 3.2 (*n* = 69)	≥ 3.2 (*n* = 27)			
*Age*					
< 57	32 (46)	19 (70)	**0.03**	–	–
≥ 57	37 (54)	8 (30)			
*Sex*					
M	29 (42)	8 (30)	0.26	–	–
F	40 (58)	19 (70)			
*BMI*					
< 24	34 (49)	13 (50)	0.78	–	–
≥ 24	30 (41)	13 (50)			
CCI					
< 1	32 (46)	6 (33)	0.14	–	–
≥ 1	37 (54)	10 (37)			
*Hinchey grade*					
0	17 (25)	6 (33)	0.8		
1a	52 (75)	21 (77)			

Variables	12-month PASS threshold		*p*	Multivariate analysis odds ratio [95% confidence interval]	*p*
	< 3.2 (*n* = 71)	≥ 3.2 (*n* = 25)			
*Age*					
< 57	35 (49)	16 (64)	0.2	–	–
≥ 57	36 (41)	9 (36)			
*Sex*					
M	28 (39)	9 (36)	0.76	–	–
F	43 (61)	16 (64)			
*BMI*					
< 24	35 (49)	12 (48)	0.62	–	–
≥ 24	30 (41)	13 (52)			
CCI					
< 1	34 (48)	15 (60)	0.29	–	–
≥ 1	37 (52)	10 (40)			
*Hinchey grade*					
0	19 (27)	4 (16)	0.27	–	–
1a	52 (73)	21 (84)			

Bold values indicate statistically significant results (*p* < 0.05)

At 1-month follow-up, unacceptable DV-QoL (PASS ≥ 3.2) was observed in 32 patients (33.3%). Univariate analysis showed that only younger age (*p* = 0.002) and a CCI < 1 (*p* = 0.01) were significantly associated with worse QoL. At the multivariable analysis, only age remained an independent predictor of unacceptable quality of life (OR 0.28, 95% CI 0.11–0.71; *p* = 0.04).

At 6 months, only younger age was significantly associated with PASS value ≥ 3.2 (*p* = 0.03) at the univariate analysis, while none of the clinical–demographic features significantly influenced QoL at the 12-month evaluation.

### Age-related distribution of DV-QoL scores (Fig. 1)

**Fig. 1:**
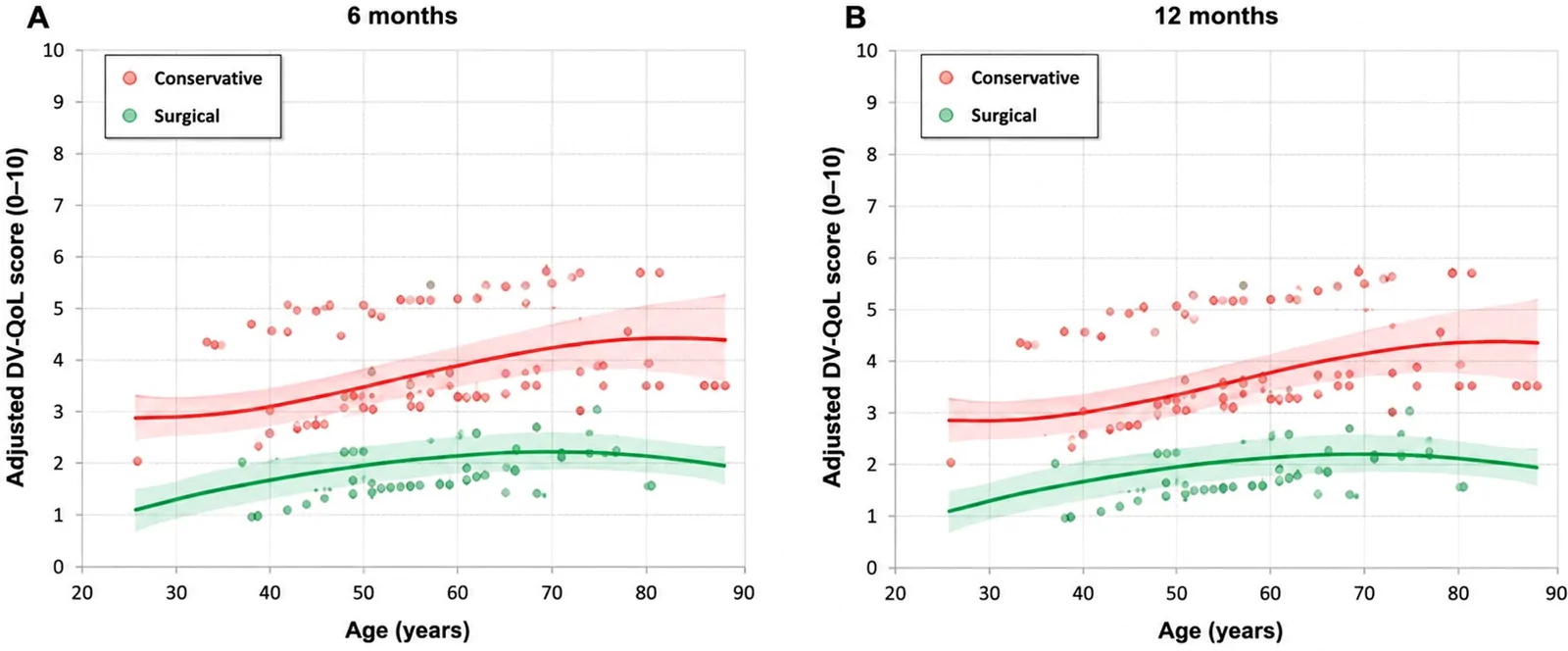
Adjusted quality of life (QoL) scores at 6 (A) and 12 (B) months, plotted against patient age for operated (green) and nonoperated (red) individuals. Solid lines represent cubic spline models illustrating the age–QoL relationship within each group. (A) Across the entire age range, operated patients exhibit better QoL scores compared with nonoperated patients, with the between-group difference being most pronounced among younger individuals (< 40 years); (B) across the entire age range, operated patients exhibit significantly higher QoL scores compared with nonoperated patients, with the between-group difference being most pronounced among younger individuals (< 40 years), and for patients older than 80 years

Scatter plots with smoothing curves were used to descriptively assess the relationship between age and DV-QoL scores at 6 and 12 months according to treatment strategy. Across the entire age range, patients managed conservatively consistently exhibited higher DV-QoL scores compared with those undergoing elective surgery, indicating worse disease-related QoL. In the nonsurgical group, DV-QoL scores showed greater dispersion and a nonlinear trend across age, with persistently elevated values at both younger and older ages. Conversely, surgically treated patients demonstrated uniformly lower DV-QoL scores with minimal variability and a relatively flat trend across age. This visual separation between groups persisted at both 6- and 12-month follow-ups, supporting the robustness and durability of the observed differences in QoL.

## Discussion

In this prospective longitudinal study, we investigated diverticulitis-specific QoL after a first episode of uncomplicated AD treated conservatively, comparing subsequent clinical surveillance versus elective laparoscopic sigmoid resection. The principal finding is that elective surgery was associated with a marked and sustained benefit in disease-related QoL: surgically treated patients exhibited near-zero DV-QoL scores at 1, 6, and 12 months, and none exceeded the PASS threshold across follow-up. Conversely, patients managed conservatively reported persistently higher DV-QoL values, with approximately one-third experiencing a non-acceptable symptom state at 1 month and roughly one-quarter still above PASS at 12 months. Overall, these results suggest that, even after a first uncomplicated episode, a clinically relevant subgroup experiences prolonged impairment that is not adequately captured by traditional endpoints such as radiologic resolution or recurrence alone. Importantly, the present findings should be interpreted within the context of the study design and should not be interpreted as evidence supporting routine elective surgery after a first uncomplicated episode, given the observational design of the study and the absence of protocolized treatment allocation. Rather, patients underwent surgery following individualized clinical assessment and shared decision-making within routine practice. Therefore, the present study was not designed to evaluate the appropriateness of surgical indications nor to compare guideline-based versus non-guideline-based management strategies. Instead, it prospectively describes QoL outcomes associated with the management pathway ultimately undertaken by patients after recovery from the index episode.

Over recent years, national and international recommendations have progressively moved away from elective resection based solely on the number of acute episodes, advocating instead for individualized decision-making after recovery from uncomplicated AD [11–15, 24]. This approach is reflected in the European Society of Coloproctology (ESCP) guidelines, which emphasize patient-centered evaluation—including symptom burden and QoL—when considering elective surgery for diverticular disease [14]. In this framework, our findings provide evidence that the impact of uncomplicated diverticulitis may extend beyond the acute inflammatory event itself, resulting in persistent symptoms, concerns, and behavioral limitations over months. This perspective supports structured follow-up strategies aimed at identifying patients who do not return to an acceptable health state despite conservative management. However, the optimal organizational model for such follow-up remains uncertain. Our findings should not be interpreted as advocating routine specialist follow-up for all patients after a first episode of acute uncomplicated diverticulitis. Rather, systematic assessment of disease-specific QoL could potentially be integrated into different models of care, including primary care, specialist outpatient clinics, or shared-care pathways, depending on local healthcare resources and organizational practices.

Our results are consistent in direction with randomized evidence comparing elective sigmoid resection and conservative management for diverticular disease, where surgery has been associated with improved QoL. The LASER randomized clinical trial reported superior QoL outcomes with elective laparoscopic sigmoid resection compared with conservative treatment in patients with recurrent, complicated, or persistent painful diverticulitis [25]. This advantage was maintained at 2-year follow-up and was accompanied by reduced recurrence rates, reinforcing the durability of the surgical benefit [26]. Similarly, the DIRECT multicenter randomized trial demonstrated that elective sigmoidectomy improved QoL compared with conservative management in patients with recurrent diverticulitis or ongoing complaints after an episode of diverticulitis, with long-term follow-up supporting sustained benefits in patient-reported outcomes [27, 28]. Although the populations of LASER and DIRECT [25–28] differ from ours—primarily including recurrent or persistent symptomatic disease rather than a “first uncomplicated episode”—their findings support the concept that elective resection may yield meaningful patient-centered benefits when diverticular disease has a significant impact on daily life. Our study extends this paradigm earlier along the disease timeline, indicating that relevant QoL impairment can occur even after the first uncomplicated episode in a sizeable proportion of patients.

A major strength of our study compared with much of the existing literature is the use of the DV-QoL questionnaire, a validated diverticulitis-specific patient-reported outcome measure designed to capture domains that are uniquely relevant to diverticular disease, including physical symptoms, concerns, emotional well-being, and behavioral changes [29]. This is particularly relevant when interpreting the literature, as several prior surgical trials have relied primarily on generic QoL tools, such as GIQLI, SF-36, and/or EQ-5D, which provide valuable global assessment but may be less sensitive to disease-specific limitations and coping behaviors typical of diverticulitis [30, 31]. In contrast, DV-QoL provides a more granular assessment of diverticulitis-related impairment and offers an additional advantage: clinically interpretable thresholds. Khor et al. established the PASS threshold (≥ 3.2) and minimal clinically important difference (MCID) for DV-QoL, allowing meaningful translation of patient-reported outcomes measure (PROM) values into patient-acceptable versus non-acceptable symptom states [23]. Within this conceptual framework, our PASS-based analysis revealed that a relevant subgroup of conservatively managed patients remained in a non-acceptable symptom state even at 12 months, highlighting a “hidden burden” that might be underestimated when focusing only on recurrence or generic QoL scores.

Importantly, the benefit of surgery in our cohort was not limited to DV-QoL total score but was consistently observed across DV-QoL domains, suggesting a multidimensional effect of elective resection. While symptom control is likely the main driver, the sustained differences in concerns, emotional well-being, and behavioral changes suggest that surgery may mitigate the broader psychosocial burden of diverticulitis, including fear of recurrence, persistent dietary restrictions, and social or occupational limitations. This is consistent with the modern patient-centered perspective of diverticular disease, in which the goal is not only preventing acute inflammatory relapses, but also restoring long-term daily functioning and well-being.

Another clinically relevant aspect is that QoL impairment in the conservative cohort persisted despite the “uncomplicated” classification of the index event. This reinforces the notion that radiologic severity alone may not reflect patient-experienced disease burden. In addition, recurrence—while important—may be an incomplete endpoint. Even in the absence of repeated acute attacks, patients may experience chronic or fluctuating gastrointestinal symptoms, ongoing restrictions, and heightened concern about future episodes. Therefore, QoL assessment should be considered a key complement to recurrence-based follow-up, particularly after an initial episode where future clinical trajectory is uncertain. In our cohort, although recurrent diverticulitis occurred in 14.6% of conservatively managed patients, only 2.1% ultimately required emergency surgery during follow-up. This relatively low risk should be carefully weighed against the morbidity associated with elective sigmoidectomy and further supports an individualized rather than routine surgical approach after a first episode of uncomplicated AD.

Regarding predictors of non-acceptable QoL after conservative management, younger age emerged as an independent predictor of PASS ≥ 3.2 at 1 month in multivariable analysis. This finding is clinically plausible: younger individuals may have greater functional expectations and lower tolerance for persistent symptoms, and they may perceive greater disruption in work, physical activity, and social life. Notably, this age-related pattern was also supported by the descriptive distribution of DV-QoL values across the age spectrum (Fig. 1). At both 6 and 12 months, conservatively managed patients consistently exhibited higher DV-QoL scores (worse disease-related QoL) with wider dispersion and a nonlinear trend, whereas surgically treated patients showed uniformly low values with minimal variability and a relatively flat trend. The persistent separation between treatment groups across the age range supports the robustness and durability of the observed QoL differences and suggests that younger patients may experience a disproportionate functional impact of post-diverticulitis symptoms and uncertainty. Overall, these findings reinforce the usefulness of early patient-reported outcomes measure (PROM)-based assessment to guide individualized counseling and management decisions.

From the surgical standpoint, postoperative outcomes in our cohort were favorable, with no conversions, no anastomotic leak, no mortality, and low morbidity limited to minor complications. This confirms that elective laparoscopic sigmoidectomy can be safely performed in selected patients within experienced centers. Nonetheless, evidence from randomized trials indicates that surgery is not without clinically relevant risk, including major complications in a subset of patients [25–28]. Therefore, shared decision-making remains essential, balancing expected QoL improvement against operative morbidity and incorporating patient priorities.

Several limitations should be acknowledged. Selection bias cannot be excluded, as the decision to proceed with surgery was based on physician judgment and patient preference. Furthermore, the management pathway adopted at our institution may not reflect routine practice in other healthcare systems, where patients with uncomplicated AD are frequently managed entirely in the outpatient setting and may not undergo specialist follow-up after the index episode. Consequently, both referral and selection bias may have influenced the study population, and the external validity of our findings should be interpreted in the context of local organizational pathways, referral patterns, and surgical expertise. Importantly, however, such bias would be expected to favor the inclusion of more symptomatic or burdened patients in the surgical group, who would typically be predisposed to worse—not better—QoL outcomes. The consistently superior QoL observed in the surgical cohort despite this potential bias may therefore reinforce the robustness of the observed association. Second, because no patient in the surgical group exceeded PASS at follow-up, complete separation prevented inclusion of treatment allocation in multivariable modeling, limiting our ability to estimate an adjusted treatment effect. Third, baseline DV-QoL referred to the month prior to the acute episode and may have been affected by recall bias. Fourth, DV-QoL was not assessed at the time treatment allocation was determined. Consequently, we were unable to compare disease-specific QoL between groups immediately before the decision to proceed with either continued conservative management or elective surgery. Finally, the study was not supported by a formal a priori sample size calculation, and patient enrollment was based on the number of consecutive eligible patients treated during the study period. Although a post hoc power analysis suggested adequate statistical power for the primary endpoint, this approach does not substitute for prospective sample size estimation and should be considered an additional limitation of the study. In addition, the duration of follow-up was limited to 1 year. Although this period was sufficient to evaluate short- and intermediate-term QoL outcomes, it may not capture late complications associated with abdominal surgery, including symptomatic incisional hernias, adhesive bowel obstruction, chronic abdominal symptoms, or the need for additional surgical interventions. Consequently, the long-term durability of the observed QoL differences between treatment strategies remains uncertain and warrants further investigation.

Despite these limitations, our data provide clinically relevant insight into a patient-centered outcome after a first episode of uncomplicated AD. They suggest that PROM-based follow-up using diverticulitis-specific instruments can identify a meaningful subgroup with persistent and unacceptable symptom burden over time. In particular, DV-QoL combined with PASS interpretation may represent a practical approach for stratifying patients early after the index episode and guiding subsequent management discussions. Although the present study was not designed to develop or validate a risk-stratification model, our findings suggest that systematic assessment of disease-specific QoL may represent a useful adjunct to clinical follow-up after a first episode of uncomplicated AD. In particular, routine use of validated PROMs such as the DV-QoL questionnaire, together with clinically interpretable thresholds such as PASS, may help identify patients with persistent impairment despite conservative management. Further prospective multicenter studies are needed to validate this approach and to define its role in future decision-making algorithms aimed at identifying patients who may derive the greatest benefit from different management strategies.

In conclusion, our findings should not be interpreted as supporting routine elective resection after a first episode of uncomplicated AD. Rather, they suggest that disease-specific PROMs may help identify patients with persistent QoL impairment after conservative management and may contribute to individualized clinical discussions. Further prospective multicenter studies are needed to validate these findings and to determine their potential implications for clinical practice.

## Supplementary Information

Below is the link to the electronic supplementary material.

Supplementary file1 (DOCX 284 kb)

## Data Availability

Data, analytic methods, and study materials are available upon reasonable request at giuseppe.quero@policlinicogemelli.it.

## Abbreviations

AD: Acute diverticulitis
ASCRS: American Society of Colon and Rectal Surgeons
BMI: Body mass index
CCI: Charlson Comorbidity Index
DV-QoL: Diverticulitis-related quality of life
ESCP: European Society of Coloproctology
ISGRC: International Study Group of Rectal Cancer
MCID: Minimal clinically important difference
ORs: Odd ratios
PASS: Patient Acceptable Symptom State
PROM: Patient-related outcomes measure
QoL: Quality of life
RCS: Restricted cubic spline
SD: Standard deviation
WSES: World Society of Emergency Surgery
