# Supplementary material for: Quality of life after conservative versus elective surgical management of first-episode uncomplicated diverticulitis: a single-center prospective study
Source: Tech Coloproctol. 2026 Aug 18;30(1):168. doi: 10.1007/s10151-026-03407-4 (PMC13759442; doi:10.1007/s10151-026-03407-4)
Supplement: Supplementary file 1 [file 10151_2026_3407_MOESM1_ESM.docx]

**Quality of life after conservative versus elective surgical management of first-episode uncomplicated diverticulitis: a single-center prospective study**

Giuseppe Quero^1,2,3^, MD, PhD; Lodovica Langellotti^1^, MD; Antonio Pio Tortorelli^1^, MD; Vincenzo Tondolo^2,4^, MD; Beatrice Biffoni^1^, MD; Davide De Sio^1^, MD; Marcello Covino^2,5^, MD; Flavia Taglioni^1^, MD; Ottavio Scrima^1^, MD; Maria Chiara Blasi^1^; Matteo Aulicino^1^, Claudio Fiorillo^1,2,3^, MD; Sergio Alfieri^1,2,3^, MD; Roberta Menghi^1,2,3^, MD

^1^ Pancreatic Surgery Unit, Fondazione Policlinico Universitario “Agostino Gemelli” IRCCS, Rome, Italy

^2^ Dipartimento di Medicina e Chirurgia Traslazionale, Università Cattolica del Sacro Cuore di Roma, Rome, Italy

^3^ Gemelli Pancreatic Center, CRMPG (Advanced Pancreatic Research Center) Fondazione Policlinico Universitario “Agostino Gemelli” IRCCS, Rome, Italy

^4^ General Surgery Unit, Fatebenefratelli Isola Tiberina—Gemelli Isola, Rome, Italy

^5^ Department of Emergency Medicine, Fondazione Policlinico Universitario Agostino Gemelli-IRCCS, 00168 Roma, Italy

**Corresponding author.**

Sergio Alfieri, MD

Pancreatic Surgery Unit

Department of Surgery, Fondazione Policlinico Universitario “Agostino Gemelli”, IRCCS

Largo Agostino Gemelli, 8

00168 Rome, Italy

tel.: +39 06 30 15 51 33

fax: +39 06 30 15 65 20

e-mail : sergio.alfieri@policlinicogemelli.it

**Supplementary Materials - Index**

| **Supplementary Figures** |  |
| --- | --- |
| Figure S1 | *pag. 3* |
| Table S1 | *pag. 4* |
|  |  |

**Supplementary Figure**

**Figure S1.** Study cohort flow diagram

| **Table S1: Longitudinal comparison of DV-QoL subdomains between the non-surgery and surgery cohorts** | | | |
| --- | --- | --- | --- |
|  | **Non-surgery group (n=96)** | **Surgery group (n=41)** | **p** |
| **One-month comparison** | | | |
| **Physical symptoms**, mean (±SD) | 5.8 (±6.09) | 1.05 (±1.43) | **< 0.001** |
| *P1 – bloated, mean (±SD)* | 1.81 (±1.94) | 0.12 (±0.45) | **< 0.001** |
| *P2 – watery stools, mean (±SD)* | 0.77 (±1.33) | 0.27 (±0.73) | **< 0.001** |
| *P3 – bowel movements, mean (±SD)* | 0.94 (±1.65) | 0.44 (±0.88) | **< 0.001** |
| *P4 – nausea, mean (±SD)* | 0.32 (±0.87) | 0.02 (±0.15) | **< 0.001** |
| *P5 – abdominal pain, mean (±SD)* | 1.68 (±1.84) | 0.20 (±0.59) | **< 0.001** |
| **Normalized physical symptoms,** mean (±SD) | 2.19 (±2.44) | 0.42 (±0.57) | **< 0.001** |
|  |  |  |  |
| **Concerns,** mean (±SD) | 4.23 (±4.71) | 0 (0) | **< 0.001** |
| *C1 – Fear of flare-up, mean (±SD)* | 1.42 (±1.73) | 0.07 (±0.34) | **< 0.001** |
| *C2 – Fear of body damage, mean (±SD)* | 1.32 (±1.52) | 0.05 (±0.32) | **< 0.001** |
| *C3 – Fear of something seriously wrong, mean (±SD)* | 1.32 (±1.52) | 0.05 (±0.32) | **< 0.001** |
| **Normalized concerns,** mean (±SD) | 3.52 (±3.92) | 0 (0) | **< 0.001** |
|  |  |  |  |
| **Feelings,** mean (±SD) | 2.82 (±4.30) | 0 (0) | **< 0.001** |
| *F1 – frustration, mean (±SD)* | 0.69 (±1.34) | 0 (0) | **< 0.001** |
| *F2 – anxiety, mean (±SD)* | 0.69 (±1.34) | 0 (0) | **< 0.001** |
| *F3 – irritation, mean (+SD)* | 0.69 (±1.34) | 0 (0) | **< 0.001** |
| *F4 – feeling looked down upon, mean (±SD)* | 0.57 (±1.07) | 0 (0) | **< 0.001** |
| **Normalized feelings,** mean (*±*SD) | 1.76 (±2.67) | 0 (0) | **< 0.001** |
|  |  |  |  |
| **Behavioral changes,** mean (±SD) | 5.03 (±4.68) | 0 (0) | **< 0.001** |
| *B1 – eat less or differently, mean (±SD)* | 2.66 (±1.34) | 0 (0) | **< 0.001** |
| *B2 – dress differently, mean (±SD)* | 0.34 (±0.8) | 0 (0) | **< 0.001** |
| *B3 – avoid social events, mean (±SD)* | 0.82 (±1.43) | 0 (0) | **< 0.001** |
| *B4 – sleep, mean (±SD)* | 0.57 (±1.07) | 0 (0) | **< 0.001** |
| *B5 – miss work or important events, mean (±SD)* | 0.63 (±1.17) | 0 (0) | **< 0.001** |
| **Normalized behavioral changes,** mean (±SD) | 2.52 (±2.34) | 0 (0) | **< 0.001** |
|  | | | |
| **Six-month comparison** | | | |
| **Physical symptoms,** mean (±SD) | 4.52 (±5.43) | 0.59 (1.08) | **< 0.001** |
| *P1 – bloated, mean (±SD)* | 1.41 (±1.75) | 0.07 (0.34) | **< 0.001** |
| *P2 – watery stools, mean (±SD)* | 0.69 (±1.34) | 0.17 (0.62) | **< 0.001** |
| *P3 – bowel movements, mean (±SD)* | 0.72 (±1.46) | 0.27 (0.66) | **< 0.001** |
| *P4 – nausea, mean (±SD)* | 0.23 (±0.78) | 0.02 (0.15) | **< 0.001** |
| *P5 – abdominal pain, mean (±SD)* | 1.45 (±1.66) | 0.05 (0.31) | **< 0.001** |
| **Normalized physical symptoms,** mean (±SD) | 1.81 (±2.17) | 0.23 (0.43) | **< 0.001** |
|  | | | |
| C**oncerns,** mean (±SD) | 3.55 (±4.28) | 0 (0) | **< 0.001** |
| *C1 – Fear of flare-up, mean (±SD)* | 1.47 (±1.25) | 0.06 (±0.24) | **< 0.001** |
| *C2 – Fear of body damage, mean (+SD)* | 1.45 (±1.25) | 0.05 (±0.12) | **< 0.001** |
| *C3 – Fear of something seriously wrong, mean (±SD)* | 1.23 (±1.19) | 0.07 (±0.22) | **< 0.001** |
| **Normalized concerns,** mean (±SD) | 2.95 (±3.56) | 0 (0) | **< 0.001** |
|  | | | |
| **Feelings,** mean (±SD) | 2.46 (±3.85) | 0 (0) | **< 0.001** |
| *F1 – frustration, mean (±SD)* | 0.55 (±1.19) | 0 (0) | **< 0.001** |
| *F2 – anxiety, mean (±SD)* | 0.69 (±1.34) | 0 (0) | **< 0.001** |
| *F3 – irritation, mean (+SD)* | 0.55 (±1.17) | 0 (0) | **< 0.001** |
| *F4 – feeling looked down upon, mean (±SD)* | 0.57 (±1.07) | 0 (0) | **< 0.001** |
| **Normalized feelings,** mean (±SD) | 1.53 (±1.39) | 0 (0) | **< 0.001** |
|  | | | |
| **Behavioral changes,** mean (±SD) | 4.21 (±3.94) | 0 (0) | **< 0.001** |
| *B1 – eat less or differently, mean (±SD)* | 2.43 (±1.45) | 0 (0) | **< 0.001** |
| *B2 – dress differently, mean (±SD)* | 0.22 (±0.71) | 0 (0) | **< 0.001** |
| *B3 – avoid social events, mean (±SD)* | 0.68 (±1.18) | 0 (0) | **< 0.001** |
| *B4 – sleep, mean (±SD)* | 0.35 (±0.89) | 0 (0) | **< 0.001** |
| *B5 – miss work or important events, mean (±SD)* | 0.42 (±0.79) | 0 (0) | **< 0.001** |
| **Normalized behavioral changes,** mean (±SD) | 2.11 (±1.97) | 0 (0) | **< 0.001** |
|  | | | |
| **Twelve-month comparison** | | | |
| **Physical symptoms,** mean (±SD) | 3.83 (±5.10) | 0.10 (±0.64) | **< 0.001** |
| *P1 – bloated, mean (±SD)* | 1.24 (±1.64) | 0.02 (±0.15) | **< 0.001** |
| *P2 – watery stools, mean (±SD)* | 0.55 (±1.16) | 0 (0) | **< 0.001** |
| *P3 – bowel movements, mean (±SD)* | 0.66 (±1.39) | 0.17 (±0.49) | **< 0.001** |
| *P4 – nausea, mean (±SD)* | 0.15 (±0.65) | 0.02 (±0.15) | **0.04** |
| *P5 – abdominal pain, mean (±SD)* | 1.24 (±1.59) | 0 (0) | **< 0.001** |
| **Normalized physical symptoms,** mean (±SD) | 1.53 (±2.04) | 0.09 (±0.25) | **< 0.001** |
|  |  |  |  |
| **Concerns,** mean (±SD) | 3.25 (±4.03) | 0 (0) | **< 0.001** |
| *C1 – Fear of flare-up, mean (±SD)* | 1.35 (±1.17) | 0.05 (±0.14) | **< 0.001** |
| *C2 – Fear of body damage, mean (+SD)* | 1.45 (±1.25) | 0.05 (±0.13) | **< 0.001** |
| *C3 – Fear of something seriously wrong, mean (±SD)* | 1.23 (±1.19) | 0.05 (±0.22) | **< 0.001** |
| **Normalized concerns,** mean (±SD) | 2.70 (±3.35) | 0 (0) | **< 0.001** |
|  |  |  |  |
| **Feelings,** mean (±SD) | 2.23 (±3.75) | 0 (0) | **< 0.001** |
| *F1 – frustration, mean (±SD)* | 0.55 (±1.19) | 0 (0) | **< 0.001** |
| *F2 – anxiety, mean (±SD)* | 0.23 (±1.05) | 0 (0) | **< 0.001** |
| *F3 – irritation, mean (+SD)* | 0.55 (±1.17) | 0 (0) | **< 0.001** |
| *F4 – feeling looked down upon, mean (±SD)* | 0.57 (±1.07) | 0 (0) | **< 0.001** |
| **Normalized feelings,** mean (±SD) | 1.38 (±2.33) | 0 (0) | **< 0.001** |
|  |  |  |  |
| **Behavioral changes,** mean (±SD) | 3.91 (±3.87) | 0 (0) | **< 0.001** |
| *B1 – eat less or differently, mean (±SD)* | 2.27 (±1.48) | 0 (0) | **< 0.001** |
| *B2 – dress differently, mean (±SD)* | 0.22 (±0.71) | 0 (0) | **< 0.001** |
| *B3 – avoid social events, mean (±SD)* | 0.60 (±1.12) | 0 (0) | **< 0.001** |
| *B4 – sleep, mean (±SD)* | 0.40 (±0.82) | 0 (0) | **< 0.001** |
| *B5 – miss work or important events, mean (±SD)* | 0.41 (±0.78) | 0 (0) | **< 0.001** |
| **Normalized behavioral changes,** mean (±SD) | 1.97 (±1.96) | 0 (0) | **< 0.001** |
